# Hand Hygiene: Knowledge and Attitudes of Fourth-Year Clerkship Medical Students at Alfaisal University, College of Medicine, Riyadh, Saudi Arabia

**DOI:** 10.7759/cureus.310

**Published:** 2015-08-24

**Authors:** Reem Hamadah, Razan Kharraz, Airabab Alshanqity, Danah AlFawaz, Abdulaziz M Eshaq, Ahmed Abu-Zaid

**Affiliations:** 1 College of Medicine, Alfaisal University

**Keywords:** hand hygiene, infection, healthcare, knowledge, attitude, clerkship, medical students, saudi arabia, medical education, curriculum

## Abstract

Introduction: Little is known about the clerkship (clinical) medical students’ knowledge of hand hygiene as the single most important precautionary measure to reduce nosocomial healthcare-associated infections. The aim of this study is to explore the knowledge of, and attitudes towards, hand hygiene practices among fourth-year clerkship medical students at Alfaisal University, College of Medicine, Riyadh, Saudi Arabia.

Materials and Methods: A cross-sectional, paper-based, Yes/No formatted questionnaire was administered to explore the students’ knowledge of, and attitudes towards, hand hygiene practices. Data were decoded in Microsoft Excel sheet and presented as numbers and percentages.

Results: One hundred and eleven students (n=111/147) participated in the questionnaire (response rate: 76%). Although the majority of students had a fair knowledge of hand hygiene practices, a number of them had some misconceptions. Only 14% of students correctly agreed to the statement: "Traditional hand washing (water, plus regular soap) decreases the number of germs." Furthermore, only 32% of students correctly answered that "hand washing with a regular soap, instead of an antiseptic soap, is better in limiting the transmission of *clostridium difficile* infections". Almost all students (93%) agreed to the importance of hand hygiene education in medical curricula and its awareness in healthcare centers. Despite the importance of hand hygiene, only 13% of students reviewed the respective WHO and CDC guidelines before starting their clinical training in the teaching hospital.

Discussion: The students’ inadequate knowledge about hand hygiene needs to be enriched by well-structured curricular and extra-curricular programs as well as more positive attitudes by healthcare workers.

## Introduction

Healthcare-associated infections (HAIs) bear huge burdens on healthcare systems. More precisely, HAIs are associated with lengthy hospitalization, long-term disability, higher microbial drug resistance, increased morbidity, greater mortality, and extra healthcare-related costs [1]. Compliance of all healthcare workers (nurses, physicians, residents, and students) to the universally agreed standard infection control precautions is identified as an effective measure to control and prevent the occurrence of HAIs [2]. These measures not merely protect patients, but the healthcare workers, too [3].

Clerkship (clinical) medical students are key players in any healthcare teams and are greatly involved in the delivery of patient care. Moreover, during their clinical training, they rotate in infection-sensitive floors, such as: labor and delivery, intensive care units, neonatal intensive care units, and operating rooms, where greater requirements of sterility and infection control are highly demanded. Despite the significant impact of HAIs on the safety and cost of healthcare systems, priority consideration of HAIs education in pre-clerkship and clerkship medical curricula has yet to be reinforced [1]. As a result, largely due to lack of knowledge and skills, clerkship students entering clinical training are at a greater risk of causing HAIs to the patients.

Hand hygiene is regarded to be the single most central precautionary measure to prevent HAIs [4]. This notion is supported in the guidelines established by the World Health Organization (WHO) [5] and Centers for Disease Control and Prevention (CDC) [6].

Little is known about the clerkship students’ knowledge of hand hygiene as one of the infection control measures. Exploring medical students’ knowledge of, and attitudes towards, hand hygiene are of high importance to public health policy makers and medical educators. Such exploration is expected to identify the curricular needs and, therefore, can be appropriately incorporated into the pre-clerkship and clerkship medical curricula to equip students with satisfactory knowledge and skills. In the short- and long-term, such curricular incorporation is expected to decrease the rate of nosocomial HAIs that could be caused by clerkship medical students. Although there are a number of reports concerning the knowledge of hand hygiene among medical students in many countries, Saudi Arabia has greatly lagged behind in this aspect [7-8].

The aim of this study is to explore the knowledge of, and attitudes towards, hand hygiene among fourth-year clerkship medical students at Alfaisal University, College of Medicine, Riyadh, Saudi Arabia.

## Materials and methods

A cross-sectional study was conducted during the fall semester of 2014-2015. The participants were fourth-year clerkship medical students enrolled in Alfaisal University, College of Medicine who were entering clinical training at a single teaching hospital in Riyadh, Saudi Arabia. This study was approved by the Institutional Review Board (IRB) of Alfaisal University (approval #2015-076).

Students were requested to complete a paper-based questionnaire. The questionnaire was administered during an academic lecture, and students were given 10 minutes to complete the questionnaire. The questionnaire was administered to explore students’ demographical data as well as their knowledge of, and attitudes towards, hand hygiene.

Demographic data included gender and previous attendance of scientific meetings (conferences, symposia, seminars, workshops, etc.) about hand hygiene. Knowledge of hand hygiene was examined by 18 Yes/No formatted questions; they were scientifically based objective questions and explored information about gloves, antiseptic soaps, and sanitizers as well as hand hygiene concepts and techniques. Attitudes towards hand hygiene were examined by five Yes/No formatted questions; they were opinion-based subjective questions and explored aspects relating to hand hygiene in terms of importance, education, compliance, and role models.

The questionnaire was developed based on a literature review (previous studies as well as WHO and CDC guidelines). All questions were peer-reviewed by two public health/infection control healthcare professionals and piloted on a group of students to ensure proper understanding and interpretation of questions.

After collecting the responses, data were decoded by using Microsoft Excel sheet (Microsoft Company, Redmond, WA, USA). Categorical data were presented as numbers and percentages.

## Results

One hundred and eleven students (n=111/147) participated in the questionnaire with an overall response rate of 76%. Around 57% and 43% of students were males and females, respectively. Around 7% of students attended previous scientific meetings about hand hygiene.

Table 1 exhibits the students’ knowledge of hand hygiene (expressed as percentages of correct answers).

**Table 1:** Students’ knowledge of hand hygiene

#	Question	Correct Answer	
		n (%)	Answer
1	Traditional hand washing (water plus regular soap) decreases the number of germs	15 (14)	Yes
2	Using gloves eliminates the necessity to wash hands	98 (88)	No
3	Hand washing is required before and after wearing gloves	80 (72)	Yes
4	Using instant hand sanitizer to quickly wash hands is always adequate	69 (62)	No
5	Must use anti-septic soap for proper hand washing	25 (23)	No
6	Must use both anti-septic soap plus hand sanitizer for proper hand washing	48 (43)	No
7	After washing hands, turn off water taps with your hands	86 (77)	No
8	After washing hands, turn off taps using piece of paper towel	71 (64)	Yes
9	Perform hand hygiene only before encountering patient (even without performing physical examination)	70 (63)	No
10	Perform hand hygiene only after encountering patient (even without performing physical examination)	92 (83)	No
11	Perform hand hygiene before and after encountering each patient (even without doing physical examination)	82 (74)	Yes
12	Enforce hand hygiene only before physically examining the patient	80 (72)	No
13	Enforce hand hygiene only after physically examining the patient	104 (94)	No
14	Enforce hand hygiene before and after physically examining the patient	81 (73)	Yes
15	Implement hand hygiene only after contact with secretions/bodily fluids (respiratory secretions, saliva, vomit and blood)	75 (68)	No
16	On unsoiled hands, an alcohol-based hand rub is recommended over an anti-septic soap hand washing	42 (38)	Yes
17	On unsoiled hands, an alcohol-based hand rub is recommended over a 3-minute surgical scrub	84 (76)	No
18	Hand washing with regular soap, instead of anti-septic soap, is better in limiting the transmission of clostridium difficile infection	36 (32)	Yes

Table 2 shows the students’ attitudes towards several aspects concerning hand hygiene.

**Table 2:** Students’ perceived attitudes towards hand hygiene

#	Question	Yes n (%)	No n (%)
1	Before starting my clinical training, I reviewed the respective WHO and CDC guidelines for hand hygiene.	14 (13)	97 (87)
2	Healthcare providers are educating patients and their families about hand hygiene and its importance.	43 (39)	68 (61)
3	I lack proper hand hygiene practices because no living examples (that is, healthcare providers) are performing them.	67 (60)	44 (40)
4	Proper hand hygiene is an important matter to be emphasized in medical curricula and healthcare centers.	103 (93)	8 (7)
5	Improper hand hygiene contributes to a patient’s morbidity and mortality.	101 (91)	10 (9)

## Discussion

Studies from Saudi Arabia that explored clerkship medical students' knowledge of hand hygiene are limited. To the best of our knowledge, there are only two related studies conducted at Qassim University, College of Medicine, Qassim, Saudi Arabia_ _[7] and King Faisal University, College of Medicine, Dammam, Saudi Arabia [8]. Our current study represents the third study so far; it beneficially contributes to the production of data generalization about clerkship students’ knowledge of hand hygiene in Saudi Arabia. The remaining 10 Saudi Arabian medical colleges are encouraged to follow in our footsteps in conducting similar studies — this is a knowledge deficit and stimulating field for further research.

Our study endeavored to examine clerkship medical students’ knowledge of, and attitudes towards, hand hygiene as one of the most important infection control measures. It revealed that students’ knowledge of hand hygiene is inadequate. Several students wrongly answered some basic hand hygiene questions. Our results were largely similar to other studies conducted elsewhere in Saudi Arabia [7-8], India [9], China [1], Namibia [10], United Kingdom [11], and Brazil  [2].

According to CDC guidelines on hand hygiene, washing hands with water and regular soap is the best available method to decrease the number of microbes on them in the vast majority of circumstances [12]. In our study, only 15 students (14%) correctly answered that "Traditional hand washing (water plus regular soap) decreases the number of germs". Conversely, in an Indian study, 88% of clerkship medical students answered this question correctly [9].

When water and soap are unavailable — which can be the case in several occasions in healthcare settings — students should look for an alternative, such as hand sanitizers. Students exhibited several major misconceptions regarding the use of hand sanitizers. This can generally be attributed to the students’ lack of knowledge. For example, 69 students (62%) falsely agreed that "Using instant hand sanitizer to quickly wash hands is adequate". Students should recognize the difference between alcohol-based and non-alcohol-based hand sanitizers. For effective killing/elimination of germs, several studies have shown that highly-concentrated alcohol-based hand sanitizers (60–95% alcohol concentration) are more superior to low-concentrated alcohol-based (less than 60% alcohol concentration) and non-alcohol-based hand sanitizers [13-14]. The disadvantages of non-alcohol-based hand sanitizers, such as skin irritation and tendency to develop resistance to the sanitizing agents, should be enlightened [13-14]. Moreover, students should recognize that alcohol-based hand sanitizers can eliminate several kinds of microbes very effectively — but only when used properly [15]. Such improper use of common hand sanitizers that preclude their antimicrobial effectiveness include an inadequate amount or rapid wiping off of sanitizer before hands have completely dried [16].

In addition, students should be aware that traditional hand washing (water, plus regular soap) has been demonstrated to be more effective at inactivating and eliminating particular kinds of germs, such as clostridium difficile-associated infections in suspected individuals/patients [17]. This is a piece of information that can be easily missed by physicians, nurses, and medical students; only 36 students (32%) answered it correctly. Likewise, in a Chinese study conducted by Huang, et al., only 23.5% of the students responded correctly [1].

Besides, students should understand that "on unsoiled hands, an alcohol-based hand rub is recommended over an antiseptic soap for proper hand hygiene", as it is quicker and better endured [14]. Only 42 students (38%) agreed correctly to this fact. Students should also recognize that hand sanitizers are infective and not recommended when hands are greatly contaminated, soiled, or greasy [14-15]. Instead, hand washing with water and regular/antiseptic soap is preferred in such conditions and remains the best modality for the control of infection spread, especially when they are visibly dirty and unclean [12].

Up to this moment, there are no well-documented and established recommendations for using both hand sanitizers and antiseptic soap to clean hands and achieve hand hygiene. Only 48 students (43%) answered this question correctly.

An infection control preventive measure that is worthy of mentioning is the use of medical gloves [2]. According to the respective WHO guidelines, 1) the use of gloves does not eliminate the necessity to wash hands and 2) hand hygiene is required before and after wearing gloves [18]. In our study, students largely showed satisfactory understanding of the two above-mentioned WHO guidelines (percentage of students’ correct answers were 88% and 72%, respectively). 

Students showed appropriate awareness of hand hygiene in terms of indications and techniques. Hand hygiene should be performed before and after each patient encounter (regardless of performing physical examination) [19], and 82 students (74%) answered this question correctly. Moreover, 81 students (73%) correctly answered that hand hygiene should be enforced before and after each physical examination. In addition, while 86 students (77%) properly disagreed to the following statement: "After washing hands, turn off water taps with your hands" [18], only 71 students (64%) properly agreed to the following statement: "After washing hands, turn off water tap using piece of paper towel" [20].

It should be noted that the relatively appropriate awareness of hand hygiene in the context of 1) patient encounter, 2) physical examination, and 3) hand washing techniques can be attributed to the intensive pre-clerkship training the students have received in the "Clinical Skills" courses at our college.

In our study, students showed positive attitudes towards hand hygiene. More than 90% of the students agreed that "Proper hand hygiene is an important matter to be emphasized in medical curricula and healthcare centers" and that "Improper hand hygiene significantly contributes to a patient’s morbidity and mortality". It is of high significance to spread the awareness about hygiene among undergraduate clerkship medical students as this will be reflected on their behaviors later on when they become professional healthcare providers. Obviously, the higher the level of education/awareness about hand hygiene standards, the more precautions will be taken, and the less ensued nosocomial HAI rates [21]. Despite the students’ realization of the significance of hand hygiene, only 14 students (13%) stated that they have reviewed the CDC and WHO guidelines for hand hygiene prior to starting their clerkship clinical training.

Sixty-seven students (60%) declared that one of the reasons accounting for their lack of proper hand hygiene is related to absence of living examples (that is, healthcare providers: physicians, residents, nurses, interns) adhering to the hand hygiene guidelines. Previous studies showed that students’ hand hygiene behaviors are substantially influenced by their role models’ (i.e., physicians) behaviors towards hand hygiene [22-23]. More specifically, in a Canadian study by Janq, et al., it was revealed that medical students’ hand hygiene were significantly affected by the hand hygiene exercised by their physicians; there was a negative impact on students’ hand hygiene as a result of non-compliance to proper hand hygiene by their senior physicians [24]. Physician role models and teamwork efforts are two valuable means to modulate and encourage proper hand hygiene by students.

Only 43 students (39%) asserted that they were "educating patients and their families about hand hygiene and its importance". This is a worrisome issue. Clerkship medical students’ roles should evolve from merely "learners" to include "public health advocators", "patient safety ambassadors", and "valuable mentors/roles models" by promoting hand hygiene compliance among patients and healthcare workers, too. These qualities should be cultivated in medical students early in pre-clerkship curricula and strongly reinforced in clerkship clinical training by their teaching physicians.

The inadequate knowledge of our students can be attributed to two main factors. First, in our pre-clerkship curriculum (as it is the case in most medical curricula in developed [11] and developing [24] countries), standard precautions, infection control measures, and hand hygiene are not sufficiently taught as independent courses or incorporated into other core courses, such as microbiology and clinical skills. Moreover, formal comprehensive reinforcing/teaching of such infection control fundamentals does not exist in the clerkship curricula either. Second, programs and campaigns, which raise awareness about hand hygiene, are largely lacking in on-campus and healthcare center settings.

There are some steps that can be taken to enhance the hand hygiene awareness among clerkship medical students. First, the formal integration of a comprehensive public health curriculum in pre-clinical (pre-clerkship) and clinical (clerkship) years is needed [25]. The significance of hand hygiene as one of the most effective infection control measures in the undergraduate curriculum should be highlighted through formal teaching and assessment, especially in clinical settings. Such a highlighting initiative is anticipated to 1) enhance students’ knowledge and 2) aid in promoting a positive culture towards hand hygiene [9]. For example, in a study conducted in Kenya, there was a remarkable advancement in knowledge about hand hygiene among school students after implementing a program about hygiene in their curriculum [26]. Nonetheless, previous studies showed that teaching infection control to medical students is challenging in terms of developing appropriate curricula and cultivating proper attitudes towards infection control [27].

Second, using the systemic Hand Hygiene Self-Assessment Framework tool can help in generating an analysis of hand hygiene of all healthcare workers at a particular healthcare center [28]. The tool works by giving information about current resources and achievements as well as identifying the improper hand hygiene that needs to be looked at and resolved. Third, organizing promotional campaigns and spreading awareness in a creative way [29] by demonstrating the importance of hand hygiene and its emphasis in the contribution to a patient’s morbidity and mortality would thus encourage its proper practice. Fourth, providing continuous feedback from mentors is useful in reinforcing the importance of hand hygiene [30].

There are a few limitations to our study. First, the study design was cross-sectional; differing results could have been obtained if another time-frame had been chosen. Second, all questions were Yes/No formatted questions, and accordingly, answers were liable to random correct guessing (without penalty for wrong answers). Third, the use of opposing statements in subsequent questions of the questionnaire may have forced respondents more towards the guessing mode for answering.

## Conclusions

Clerkship medical students had some misconceptions about hand hygiene that reflected their inadequate knowledge. Medical educators should design well-structured pre-clerkship and clerkship curricula that guarantee satisfactory knowledge of infection control measures and hand hygiene. Public awareness and creative campaigns about hand hygiene are also encouraged. Developing positive attitudes towards hand hygiene and the students’ compliance to such should be underscored as being essential to clinical practice and reducing the rate of HAIs. Incorporating formal curricular assessments is one plausible method to cultivate this compliance. Students’ adequate knowledge of hand hygiene is expected to decrease the rate of nosocomial HAIs that could be incurred unintentionally or negligibly by the clerkship medical students. Future research projects include exploring students’ perception on barriers towards hand hygiene education and practice as well as exploring the impact of mentoring role models (physicians, nurses, etc.) towards proper hand hygiene.
